# Diurnal fluctuations in the content of soluble sugars and the expression of the TAI and LIN6 invertase genes and the STP1 sugar transporter gene in the leaves of the tomato (Solanum lycopersicum L.)

**DOI:** 10.18699/vjgb-25-07

**Published:** 2025-02

**Authors:** M.A. Filyushin, A.V. Shchennikova, E.Z. Kochieva

**Affiliations:** Federal Research Center “Fundamentals of Biotechnology” of the Russian Academy of Sciences, Moscow, Russia; Federal Research Center “Fundamentals of Biotechnology” of the Russian Academy of Sciences, Moscow, Russia; Federal Research Center “Fundamentals of Biotechnology” of the Russian Academy of Sciences, Moscow, Russia

**Keywords:** tomato, Solanum lycopersicum L., soluble sugars, invertases, hexose transporter, gene expression, circadian rhythm, томат, Solanum lycopersicum L., растворимые сахара, инвертазы, транспортер гексоз, экспрессия генов, циркадный ритм

## Abstract

The content of hexoses (fructose, glucose) essential for the fruit of the tomato (Solanum lycopersicum L.) is regulated by the joint activity of sucrose hydrolysis enzymes (including invertases), invertase inhibitors, and sugar transporters. In addition to fruit taste, soluble sugars are closely related to the stress resistance of the tomato plant. In this work, we determined the diurnal dynamics of the content of soluble sugars (sucrose, fructose and glucose) and the expression of genes for sucrose hydrolysis enzymes (vacuolar invertase TAI, cell wall invertase LIN6) and the hexose transporter (STP1) in the leaves of the tomato variety Korneevsky. It was shown that both the amount of sugars and the level of transcripts of the TAI, LIN6 and STP1 genes depend on the circadian rhythm and correspond to the biological processes occurring in the plant at different periods of the day. The content of sucrose and hexoses changes in a similar way during the day. At the beginning of the light phase, the concentration of sugars is minimal, at the end it has the highest daily values; at the beginning of the dark phase, it shows a residual increase and then decreases towards the end of the phase. In silico analysis of organ-specific expression of TAI, LIN6 and STP1 in S. lycopersicum cv. Micro-Tom showed the presence of mRNA of all three genes in all tissues. The TAI gene was expressed most strongly in ripe fruits, while the level of LIN6 and STP1 transcripts was extremely low. The level of TAI mRNA in the leaves was ~2 times higher than that of LIN6 and ~27 times higher than that of STP1. Analysis using qRT-PCR of the diurnal dynamics of TAI, LIN6 and STP1 expression in the cv. Korneevsky leaves showed that all three genes were expressed at all points analyzed. Fluctuations in their expression levels occur in a similar manner: mRNA levels reach peak values in the middle of the light and dark phases. The results obtained are important for understanding the functions of invertases and sugar transporters in the tomato plant, and can be used in predicting the stress resistance of plants in tomato breeding.

## Abstract (ru)

Содержание основных для плода томата (Solanum lycopersicum L.) гексоз (фруктозы, глюкозы) регулируется совместной активностью ферментов гидролиза сахарозы (включая инвертазы), ингибиторов инвертаз и транспортеров сахаров. Кроме вкусовых качеств, растворимые сахара тесно связаны со стрессоустойчивостью растений томата. В настоящей работе была определена суточная динамика содержания растворимых сахаров (сахарозы, фруктозы и глюкозы), а также экспрессия генов ферментов гидролиза сахарозы (вакуолярной инвертазы TAI, инвертазы клеточной стенки LIN6) и транспортера гексоз (STP1) в листьях растений томата сорта Корнеевский. Было показано, что количество сахаров и уровень транскриптов генов TAI, LIN6 и STP1 зависят от циркадного ритма и соответствуют биологическим процессам, протекающим в растении в разное время суток. Содержание сахарозы и гексоз в течение суток изменяется сходным образом. В начале световой фазы концентрация сахаров минимальна, в конце – имеет наибольшие дневные значения; в начале темновой фазы демонстрирует остаточный рост и затем снижается к концу фазы. Анализ in silico органоспецифичной экспрессии TAI, LIN6 и STP1 у сорта Micro-Tom S. lycopersicum показал наличие их транскриптов во всех тканях. Ген TAI экспрессировался наиболее активно в спелых плодах, тогда как уровень транскриптов LIN6 и STP1 в этих органах носил следовой характер. В листьях уровень мРНК TAI был выше, чем таковой LIN6 и STP1, в ~2 и ~27 раз соответственно. Анализ с помощью ПЦР-РВ суточной динамики экспрессии генов TAI, LIN6 и STP1 в листьях растений сорта Корнеевский показал, что гены экспрессируются во всех проанализированных временных точках. Колебания уровня экспрессии генов происходят сходным образом: уровень мРНК достигает пиковых значений в середине световой и темновой фаз. Полученные результаты важны для понимания функций инвертаз и транспортеров сахаров в растении томата и могут быть использованы в селекции при прогнозировании стрессоустойчивости растений

## Introduction

During photosynthesis, the plant accumulates assimilates – vital organic compounds utilized for respiration, maintenance of cell metabolism, growth and development. The sucrose is the main transport form of photoassimilates during distribution throughout the plant (Lemoine et al., 2013). The signals for distribution are provided by sucrose and glucose molecules, the number of which influences the regulation of genes active at a particular stage of plant development (Koch, 2004; González et al., 2005; Rolland et al., 2006).

After delivery to storage organs (flowers, fruits, tubers, etc.), sucrose is utilized by being broken down into glucose and fructose by sucrose synthases (reversible hydrolysis) or invertases (irreversible hydrolysis); the functions of the latter are highly variable and closely related to localization in different cellular compartments (Roitsch, González, 2004). The expression level of invertase genes depends on the type of tissue/organ, stage of plant development, and external stimuli, including exposure to stress factors, phytohormones, elicitors, etc. (Roitsch, González, 2004; Koch, 2004; Proels, Roitsch, 2009). Cell wall invertases are involved in the distribution of sucrose in plant tissues and organs and signal transduction, while vacuolar invertases are involved in sugar accumulation and osmoregulation (Roitsch, González, 2004; González et al., 2005). Hexoses formed during sucrose hydrolysis enter the cells of storage tissues via hexose transporters (Proels, Roitsch, 2009).

Tomato (Solanum lycopersicum L.) is one of the most popular vegetable crops in the world. Tomato fruits accumulate glucose and fructose during ripening (Beckles et al., 2012). These hexoses affect the degree of fruit sweetness, and their amount is regulated by the combined activity of sucrose synthases (reversible hydrolysis of sucrose), invertases (irreversible hydrolysis of sucrose), invertase inhibitors, and sugar transporters (Kawaguchi et al., 2021; Wang B. et al., 2021). In addition to determining an important fruit quality trait, soluble sugars significantly contribute to the regulation of stress resistance of tomato plants during growth and development (Proels, Roitsch, 2009). Increased carbohydrate influx to the stressed area provides energy for protective reactions, stimulation of carbohydrate accumulation and modulation of the expression of the corresponding genes, including genes for invertases and sugar transporters (Proels, Roitsch, 2009). The coordinated induction of the monosaccharide transporter and cell wall invertase genes observed under biotic stress (Fotopoulos et al., 2003; Voegele et al., 2006) supports the important role of apoplastic sucrose degradation in mediating defense responses. Regardless of the process, both the metabolism and distribution of sugars, and, thus, the expression of the genes involved, are controlled by circadian rhythms, in particular diurnal variations in the intensity of biological processes (González et al., 2005; Rolland et al., 2006).

Among tomato invertases, the most significant roles belong to the cell wall invertase LIN6 (Wiv-1) (Proels, Roitsch, 2009) and the vacuolar invertase TAI (other names AI, PAIN1) (Elliott et al., 1993). The LIN6 enzyme is important for plant growth and response to various stress factors, and is also under the control of key circadian oscillator factors (Proels, Roitsch, 2009; Zhang et al., 2013). TAI activity is associated mainly with sucrose hydrolysis in the tomato fruit (Slugina et al., 2017). No data on the possible dependence of TAI gene expression on circadian rhythms in tomato plants have been found, but the dependence is assumed, since it has been shown using the example of vacuolar invertase of sugar beet Beta vulgaris (González et al., 2005).

Hexose transporters in tomato include the most well-known proteins STP1 and STP2. Knockdown of the genes encoding them reduces the amount of glucose and fructose in the roots, which reduces the plant’s sensitivity to nematode infestation (Warnock et al., 2016). Of particular note is the STP1 gene, which is considered a target of domestication in the tomato genome; lack of STP1 expression negatively affects the efficiency of fruiting and the amount of sugars in the fruit (Wang Y. et al., 2023). The available literature does not mention the presence of a dependence of STP expression on the circadian oscillator; however, a connection is assumed, as for invertases.

In this study, we analyzed the dependence of the expression levels of vacuolar invertase TAI, cell wall invertase LIN6 and hexose transporter STP1 genes, as well as the content of soluble sugars (sucrose, glucose, fructose), on the diurnal rhythm during tomato plant growth. The results obtained are important for understanding the functions of invertases and sugar transporters in tomato plants.

## Materials and methods

The study was carried out on cv. Korneevsky S. lycopersicum, bred at the Federal Scientific Vegetable Center (FSVC, Moscow Region, Russia). The cultivar is mid-season, suitable for greenhouse conditions, produces fruits with high sugar content, and is resistant to various stress factors, including fluctuations in temperature and photoperiod (accession number 8262334, https://gossortrf.ru/registry/).

Tomato plants of cv. Korneevsky were grown to the fruiting stage in 2023 in greenhouse conditions of the FSVC. The collected seeds were used (2024) to obtain seedlings at the 5–7 leaf phase (experimental climate control facility, Federal Research Center of Biotechnology, Russian Academy of Sciences) under conditions of a long photoperiod and optimal temperature (day/night – 16 h/8 h, 23 °C/21 °C; light phase from 7:00 to 23:00; illumination 190 μM/(m2·s)). Leaf samples (two plants for each analysis point) were collected during the day at six time points: 1 h before (6:00) and after (8:00) the onset of the light phase; in the middle of the light phase (15:00); 1 h before (22:00) and after (24:00) the onset of the dark phase; in the middle of the dark phase (3:00). The tissue was ground in liquid nitrogen and used for analysis of the content of soluble sugars and the expression level of invertase (TAI, Solyc03g083910; LIN6, Solyc10g083290) and hexose transporter (STP1, Solyc02g079220) genes.

The concentration (mg/100 g of fresh weight (FW)) of soluble sugars (sucrose, glucose, fructose) was determined using the Enzytec™ Liquid Sucrose/D-Glucose and Enzytec ™ Liquid D-Glucose/D-Fructose tests (R-Biopharm AG, Germany).

A preliminary profiling of TAI, LIN6, and STP1 expression in different tomato plant organs was performed in silico using transcriptome data for the cv. Micro-Tom S. lycopersicum (TomExpress database; http://tomexpress.toulouse.inra.fr/ login) (Zouine et al., 2017). Data were visualized using online HeatMapper (http://www2.heatmapper.ca/expression/) based on FPKM (Fragments per kilobase of transcript per million mapped fragments; TomExpress) values.

To analyze gene expression using quantitative real-time PCR (qRT-PCR), total RNA was isolated from 0.2–0.5 g of collected leaf material and purified from DNA impurities (RNeasy Plant Mini Kit, RNase-free DNase set, QIAGEN, Germany). Based on total RNA preps, cDNA was synthesized (GoScript Reverse Transcription System, Promega, USA). The concentration of RNA and cDNA preparations was determined fluometrically (Qubit® Fluorometer, Thermo Fisher Scientific, USA; Qubit RNA HS Assay Kit, Invitrogen, USA). Primers for qRT-PCR were designed by structural analysis of the S. lycopersicum genes and their transcripts (available in the databases: https://www.ncbi.nlm.nih.gov/; https://solgenomics. net/) using NCBI-BLAST (https://blast.ncbi.nlm.nih.gov/ Blast.cgi) and MEGA 7.0 (https://www.megasoftware.net/). Primers for qRT-PCR were designed for the LIN6 (5′-ttcc gatgcctcaaggtcaag-3′, 5′-cacgtttttcctccagcacca-3′) and STP1 (5′-tgctcagaatgttgctatgctc-3′, 5′-gtgctcctctgtatttgtatgg-3′) genes. For the TAI gene, we used the primers developed earlier (Slugina et al., 2017). The qRT-PCR reaction mixture included cDNA (3 ng), specific primers and “2.5 × Reaction mixture for qRT-PCR in the presence of SYBR Green I and ROX” (Synthol LLC, Russia). qRT-PCR was performed in a CFX96 Real-Time PCR Detection System (Bio-Rad Laboratories, USA); program: 5 min, 95 °C, 40 cycles (15 s, 95 °C; 40 s, 60 °C). Data were normalized to the expression of two reference genes, Expressed (SGN-U346908) and actin2/7 (NM_001330119.1) (Efremov et al., 2020). The analysis was carried out in two biological and three technical replicates.

The results of the analysis of sugar content (mg/100 g FW) and gene expression were statistically processed using GraphPad Prism v. 8 (GraphPad Software Inc., USA; https://www.graphpad.com/scientific-software/prism/). The significance (p < 0.05) of differences between the values obtained for the time points was determined using Two-way ANOVA (“multiple comparisons, corrected with the Bonferroni test”).

## Results

In this study, using the cv. Korneevsky tomato (S. lycopersicum) as an example, we characterized daily changes in the content of soluble sugars, as well as the expression pattern of genes of two key invertases (vacuolar, TAI; cell wall, LIN6) and sugar transporter (STP1) in the leaves of a plant in the active stage of vegetative growth and development (5–7 leaves).

Since S. lycopersicum is day-neutral species (Lifschitz, Eshed, 2006), a long photoperiod (16 h/8 h – day/night), typical for summer, was used in the work. Time points for measuring the target indicators were selected considering the boundaries between daily phases (one hour before and after the onset of light (points 6:00, 8:00) and darkness (22:00, 24:00)), as well as the middle of the dark (3:00) and light (15:00) periods.

**Fig. 1.**
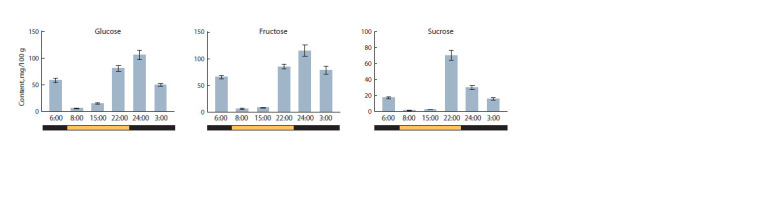
Diurnal dynamics of the content (mg/100 g FW) of glucose, fructose and sucrose in the leaves of a cv. Korneevsky tomato plant (S. lycopersicum). The values of sugar concentration at the analyzed time points differ (p <0.05), with the exception of glucose (6:00 vs. 3:00; 8:00 vs. 15:00), fructose (6:00 vs. 22:00; 8:00 vs. 15:00; 6:00 vs. 3:00; 22:00 vs. 3:00), and sucrose (6:00 vs. 3:00; 8:00 vs. 15:00).

At these six points, the content of soluble sugars (glucose, fructose and sucrose) was measured (Fig. 1). It was shown that the amount of all analyzed sugars is minimal at the beginning of the light phase. By the middle of the day (15:00) it increases by ~1.2–2.0 times, and by the end of the photoperiod (22:00), it sharply rises by ~15 times (vs. 8:00), reaching the daily maximum. At the beginning of the dark phase (24:00), the content of hexoses continues to grow (by ~1.3–1.6 times vs. 22:00); however, in the second half of the dark period (3:00, 6:00), it decreases (by ~1.5–2.0 times vs. 24:00). At the beginning of the light phase (8:00), the number of hexoses decreases even more sharply (by ~50–60 times vs. 6:00) (Fig. 1).

The sucrose content changes during the day in a manner similar to that of hexoses, with the exception of the 24:00 point (a decrease of ~2 times vs. 22:00) and a smoother decrease compared to hexoses at the 8:00 point (~18 times vs. 6:00) (Fig. 1).

Thus, it was shown that the content of the analyzed soluble sugars is minimal at the beginning and maximal at the end of the photoperiod, while in the dark phase their amount is more constant

**Fig. 2.**
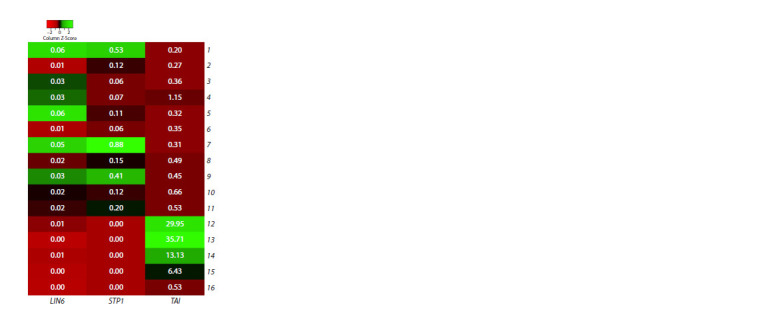
Graphical visualization (heat map) of TAI, LIN6, and STP1 gene expression data in cv. Micro-Tom tomato (S. lycopersicum) constructed using TomExpress transcriptome data (Zouine et al., 2017). Organs analyzed: root (1); leaf (2); bud (3); flower at the anthesis stage (4); fruit, 4 days post anthesis (dpa) (5); pulp (6) and skin (7) of the fruit (10 dpa); pulp (8) and skin (9) of the fruit (35 dpa); pulp (10) and skin (11) of the fruit (38 dpa, BR); pulp (12) and skin (13) of the fruit (41 dpa, OR); pulp (14) and skin (15) of the fruit (44 dpa, RR); mature seeds (16). The rectangles show FPKM values rounded to the second decimal place.

Next, the expression pattern of the TAI, LIN6, and STP1 genes was characterized. A preliminary analysis of the organspecific expression pattern of these genes was performed in silico (Fig. 2). It was shown that transcripts of all three genes are present in vegetative tissues and in the growing fruit (including the breaker (BR) stage of reaching the final size and the beginning of the color change). In the ripening fruit (the orange (OR) and red ripe (RR) fruit stages), only trace numbers of LIN6 and STP1 transcripts (0.002–0.0129 FPKM) were detected.

At the same time, TAI was expressed most intensely in these tissues. The peak of TAI expression (26.95–35.71 FPKM) corresponded to the OR stage of fruit ripening, where the level of gene transcripts was ~2 and ~27–36 times higher than in the fruit at the RR stage (6.43–13.13 FPKM) and in vegetative tissues/ growing fruit (including the BR stage; 0.20–1.15 FPKM),respectively. In other reproductive tissues – buds, flowers and RR fruit seeds, the number of TAI transcripts was ~6–12, 20–40 and 50 times higher, respectively, compared to LIN6 and STP1 (Fig. 2). 

Despite the obvious specificity of TAI activity to the ripe fruit, its expression level in vegetative organs (0.20– 1.15 FPKM) was, on average, higher than that of LIN6 (0.01–0.06 FPKM) and STP1 (0.06–0.88 FPKM). In whole leaf tissue, the number of TAI transcripts was ~2 and ~27 times higher than that of LIN6 and STP1, respectively. The LIN6 expression level in the leaves was the lowest (~12 times lower than STP1) (Fig. 2).

Thus, in silico profiling of gene expression showed that in all plant organs, the activity of the vacuolar invertase gene TAI significantly exceeds that of the cell wall invertase gene LIN6 and the sugar transporter gene STP1. Moreover, TAI expression is highest in the reproductive organs, especially in the storage tissues of the ripe fruit at the OR and RR stages

**Fig. 3.**
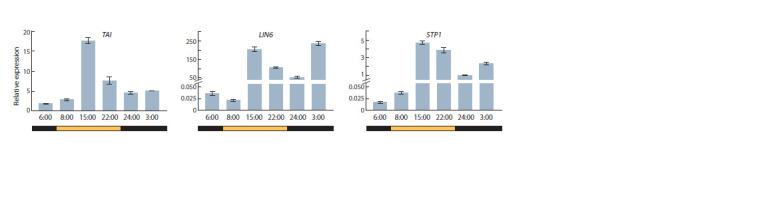
Expression patterns of the TAI, LIN6 and STP1 genes based on qRT-PCR data. The relative transcript levels for each gene at the analyzed time points differ significantly (p < 0.05), with the exception of TAI (6:00 vs. 8:00; 24:00 vs. 3:00), LIN6 (6:00 vs. 8:00), and STP1 (6:00 vs. 8:00).

Next, in the same leaf samples of cv. Korneevsky used for the analysis of sugar content, the expression levels of the TAI, LIN6 and STP1 genes were determined (using qRT-PCR) at six time points during the day. As a result, it was shown that all three genes are expressed at all six time points. On average, the highest relative transcript levels were observed for LIN6, and the lowest, for STP1. The expression level of TAI (contrary to expectations based on in silico data, Fig. 2) was an order of magnitude lower than that of LIN6, and only ~3–4 times higher than the level of STP1 transcripts (Fig. 3).

Overall, the change in the diurnal expression dynamics was similar for all three genes: TAI, LIN6 and STP1. The transcript level increased significantly (by ~7 (TAI), ~7,000 (LIN6) and ~128 (STP1) times) from the 8:00 to the 15:00 point of the light phase. Then it decreased less sharply towards its end (~1.2–2.5 times, 22:00 vs. 15:00) and the beginning of the dark phase (~2–3.5 times, 24:00 vs. 22:00). By the middle of the night, the gene expression was upregulated (by ~2–5 times, 3:00 vs. 24:00), and by the end of the dark phase, it decreased by ~2.5 (TAI), ~7,000 (LIN6), and ~183 (STP1) times (6:00 vs. 3:00) (Fig. 3).

Thus, the diurnal dynamics of the TAI, LIN6 and STP1 expression level was similar, but fluctuations in the case of LIN6 and STP1 were significantly more pronounced than those of the TAI gene. Nevertheless, the pre-dawn and early afternoon expression level of LIN6 and STP1 was extremely low, while that of TAI was relatively constant and notable.

## Discussion

Soluble mono- and disaccharides have a significant impact on plant growth and development (Proels, Roitsch, 2009; Lemoine et al., 2013). Their content is regulated (besides glycan synthesis/degradation) through sucrose hydrolysis by sucrose synthases, invertases and invertase inhibitors, as well as through transfer between tissues using transporters (Kawaguchi et al., 2021; Wang B. et al., 2021)

Soluble sugars play an important role in all developmental processes in plant species, including tomato plants (Proels, Roitsch, 2009). Moreover, under stress, the influx of carbohydrates to the affected areas increases, which provides energy for a protective response, including the coordinated stimulation of carbohydrate accumulation and the expression of invertase and sugar transporter genes (Fotopoulos et al., 2003; Voegele et al., 2006; Proels, Roitsch, 2009; Bolouri Moghaddam, Van den Ende, 2013)

In any process occurring in a plant involving soluble sugars, both the sugar content and the intensity of expression of the corresponding genes are characterized by synchronous cyclical oscillations during the day under the control of a circadian oscillator (González et al., 2005; Rolland et al., 2006).

In this study, we analyzed the diurnal dynamics of the concentration of soluble sugars (sucrose, glucose, and fructose) in the leaves of cv. Korneevsky tomato seedlings. The measurement points covered the border periods between the dark and light phases (6:00, 8:00, 22:00, 24:00) and the middle of the phases (15:00, 3:00). At the same points, we determined the expression of the genes of vacuolar invertase (TAI), cell wall invertase (LIN6), and hexose transporter (STP1), the role of which in tomato sugar metabolism is most important (Elliott et al., 1993; Proels, Roitsch, 2009; Warnock et al., 2016; Slugina et al., 2017).

The resulting diurnal profile of sugar content (Fig. 1) is consistent with known active sucrose synthesis in the daytime phase of photosynthesis, as well as with the diurnal cycle of sugar accumulation/intake due to the day/night synthesis/ degradation of transient starch (Haydon et al., 2011). During the light phase, sucrose, glucose and fructose gradually accumulate (Fig. 1). Some of the glucose is presumably utilized for the synthesis of transient starch, and at the same time, sugars are released from the leaves (as sources of sugars) into storage organs (in our case, into the roots of the seedlings). By the end of the day, the amount of sugars reaches its highest values, and in the dark phase it tends to decrease and then is maintained at a more or less constant level (Fig. 1), due to the pause in sucrose synthesis and the activation of the transient starch degradation (Koch, 2004; Haydon et al., 2011).

The main result of the in silico characterization of TAI, LIN6, and STP1 expression (Fig. 2) is the confirmation of the important role of the vacuolar invertase gene TAI in sucrose hydrolysis in the ripe fruit as a storage organ, shown earlier (Elliott et al., 1993; Slugina et al., 2017). Furthermore, the higher transcript level of TAI compared to LIN6 (Fig. 2) assumes a greater importance of TAI (than that of LIN6) for sucrose hydrolysis in vegetative tissue as well. Nevertheless, the significant transcript number of the cell wall invertase gene LIN6 in vegetative tissues indicates the known importance of LIN6 for plant vegetative growth (Proels, Roitsch, 2009; Zhang et al., 2013). Also, the presence of transcripts of the LIN6 gene and the sugar transporter gene STP1 in vegetative tissues (Fig. 2) is consistent with the previously shown involvement of these genes in the plant stress response (Proels, Roitsch, 2009; Warnock et al., 2016). At the same time, the extremely low number of STP1 transcripts in ripe fruits of cv. Micro-Tom (Fig. 2), given the high sugar content in the fruits of this cultivar and the shown direct relationship between the expression level of this gene and the amount of sugars in the fruits (Wang Y. et al., 2023), suggests that even low activity of the STP1 gene is sufficient to implement this relationship.

Subsequent analysis of the diurnal dynamics of TAI, LIN6, and STP1 expression in the leaves of cv. Korneevsky plants showed that the levels of all three genes change in a similar manner and in association with the circadian rhythm (Fig. 3). The diurnal dynamics of TAI expression is consistent with the previously demonstrated diurnal dynamics for the B. vulgaris vacuolar invertase gene (González et al., 2005): both genes reach peak expression in the middle of the light phase.

Unlike TAI, the cell wall invertase gene LIN6 has another expression maximum – in the middle of the night (Fig. 3). Moreover, contrary to in silico data (Fig. 2), the level of LIN6 transcripts was an order of magnitude higher than that of TAI (Fig. 3). A possible reason for the discrepancy may be older plants (compared to our study) and, therefore, older leaves with large vacuoles in the cells taken into the transcriptome analysis, the results of which are presented in the in silico database we used (Zouine et al., 2017). We tested seedlings at the 5–7 leaf stage, the young leaves of which contained small vacuoles in the cells, which suggests more active apoplastic processes of sucrose hydrolysis and sugar transport. This is also supported by significantly more pronounced (by an order of magnitude) diurnal fluctuations in the expression of LIN6 and STP1 compared to TAI (Fig. 3).

## Conclusion

In this study, the diurnal dynamics of the content of soluble sugars and the expression of genes encoding sucrose hydrolysis enzymes (invertase genes TAI and LIN6) and sugar transfer proteins (STP1 transporter gene) in tomato seedlings of cv. Korneevsky was determined. It was shown that both sugars and the transcript level of TAI, LIN6 and STP1 depend on the circadian rhythm and correspond to biological processes occurring in the plant at different periods of the day. The results obtained are important for understanding the functions of invertases and sugar transporters in the tomato plant, and can be used to predict plant stress resistance in tomato breeding.

## Conflict of interest

The authors declare no conflict of interest.
